# Autistic Traits and Emotional Well-Being in First-Year University Students

**DOI:** 10.1192/j.eurpsy.2026.10457

**Published:** 2026-07-31

**Authors:** È. Pagespetit, M. Pagerols, R. Prat, J. Puigbó, M. Andreu, G. Español-Martín, M. Casas, R. Bosch

**Affiliations:** 1 Programa MIND Escoles, Sant Joan de Déu Research Institute, Esplugues de Llobregat; 2 Faculty of Medicine, University of Vic – Central University of Catalonia, Vic; 3 CIBERSAM, Instituto de Salud Carlos III, Madrid; 4 Unitat de Farmacologia, Departament de Fonaments Clínics, Facultat de Medicina i Ciències de la Salut, Universitat de Barcelona; 5 Faculty of Psychology and Education Sciences, Open University of Catalonia; 6 Department of Psychiatry, Hospital Universitari Vall d’Hebron; 7 Department of Psychiatry and Forensic Medicine, Universitat Autònoma de Barcelona; 8 Fundació d’Investigació Salut i Progrés; 9 Instituto para el Desarrollo de Terapias Avanzadas en Neurociencias, Barcelona; 10 Divisió de Salut Mental, Althaia Xarxa Assistencial Universitària de Manresa, Manresa, Spain

## Abstract

**Introduction:**

Autism Spectrum Disorder (ASD) influences the social and emotional adaptation of university students, a group increasingly exposed to academic and emotional demands.

**Objectives:**

This study analyzed the influence of gender, field of study and psychopathological symptoms on autistic traits, and the link between autistic traits and emotional well-being in first-year university students.

**Methods:**

The sample comprised 1,373 students (57.2% women, 42.8% men) aged 18–21 from 14 faculties of two Spanish universities. Data were collected during supervised classroom sessions using online questionnaires, which included sociodemographic information and four validated scales (i.e., Autism-Spectrum Quotient–Short Version (AQ-Short), Adult Self-Report, ADHD Rating Scale, World Health Organization-Five Well-Being Index). Statistical analyses included t-tests, ANOVA, correlations, and linear regression.

**Results:**

Differences in ASD traits were found by field of study (*p*<.001). Students of science, technology, engineering, and mathematics (STEM) scored higher on the AQ-Short total score (M=62.3, SD=9.53) and the Social Skills subscale (*M*=14.9, *SD*=4.22) compared to students in Social Sciences (*M*=59.5, *SD*=8.15 and *M*=13.7, *SD*=3.80, respectively). They also scored higher on the Imagination subscale (*M*=16.4, *SD*=3.55) than students in Health Sciences (*M*=15.5, *SD*=3.67), and on the Numbers and Patterns subscale (*M*=12.1, *SD*=3.30) compared to all other fields of study (Social Sciences: *M*=11.0, *SD*=3.12; Health Sciences: *M*=10.9, *SD*=3.22; Humanities: *M*=10.5, *SD*=3.21). Similarly, gender differences were observed across the AQ-Short subscales, with women scoring higher on Social Skills (women: *M*=14.5, *SD*=4.23; men: *M*=14.0, *SD*=3.98) and Routine (women: *M*=10.2, *SD*=2.33; men: *M*=9.50, *SD*=2.10), while men scored higher on Numbers and Patterns (women: *M*=11.0, *SD*=3.28; men: *M*=11.9, *SD*=3.16). ASD traits positively correlated with ADHD symptoms (r=.294, *p*<.001), internalizing (r=.551, *p*<.001) and externalizing problems (r=.155, *p*<.001). Likewise, all AQ-Short subscales were associated with these psychopathological dimensions, except for Social Skills and Imagination, which did not correlate with externalizing problems. Regression analysis showed that higher ASD traits predicted lower emotional well-being (*β*=-0.069, *p*=.013) even after adjusting for field of study, gender, ADHD, and psychopathological symptoms. The regression model explained 42,5% of the variance in emotional well-being.

**Conclusions:**

Higher autistic traits were linked to other psychopathological symptoms and lower emotional well-being, suggesting that the AQ-Short may capture not only ASD-related features but also broader emotional and adaptive difficulties, which are especially relevant in first-year university populations.

**Disclosure of Interest:**

È. Pagespetit: None Declared, M. Pagerols: None Declared, R. Prat: None Declared, J. Puigbó: None Declared, M. Andreu: None Declared, G. Español-Martín Grant / Research support from: Has received unrestricted educational and research support from the following companies during the last 3 years: Exeltis, Idorsia, Janssen-Cilag, Neuraxpharm, Oryzon, Roche, Probitas, Psious, and Rubió., M. Casas Speakers bureau of: Has received fees from Takeda and Laboratorios Rubió for participation in psychiatric meetings., R. Bosch: None Declared

